# Anti-*Leishmania infantum* Antibody-Producing Plasma Cells in the Spleen in Canine Visceral Leishmaniasis

**DOI:** 10.3390/pathogens10121635

**Published:** 2021-12-17

**Authors:** Jonathan L. M. Fontes, Bianca R. Mesquita, Reginaldo Brito, Juliana C. S. Gomes, Caroline V. B. de Melo, Washington L. C. dos Santos

**Affiliations:** Laboratório de Patologia Estrutural e Molecular, Instituto Gonçalo Moniz, Fiocruz-BA, Salvador 40296710, Brazil; jonathan.fontes@fiocruz.br (J.L.M.F.); bianca.mesquita@fiocruz.br (B.R.M.); reginaldo.brito@fiocruz.br (R.B.); gomes.juliana@ufba.br (J.C.S.G.); caroline.melo@rivm.nl (C.V.B.d.M.)

**Keywords:** visceral leishmaniasis, spleen, plasma cell, plasmacytosis, antibody

## Abstract

The spleen is involved in visceral leishmaniasis immunopathogenesis, and presents alterations in white-pulp microenvironments that are associated with an increased susceptibility to coinfections and patient death. Plasmacytosis in splenic red pulp (RP) is one observed alteration, but the specificity of antibody-secreting cells and the distribution of them has not yet been evaluated. We biotinylated soluble *L. infantum* membrane antigens (bSLMA) used as probes in modified immunohistochemistry, and detected the presence of anti-*L. infantum* antibody-secreting cells. Were used spleens from eight dogs from the endemic area for canine visceral leishmaniasis (CanL), and three healthier controls. The spleen sections were cryopreserved, and we performed modified immunohistochemistry. The ratio of plasma cells which were reactive to bSLMA (Anti-Leish-PC) in the spleen RP and periarteriolar lymphatic sheath (PALS) were calculated. Dogs with CanL present hyperglobulinemia and more plasma cells in their RP than the controls. Furthermore, dogs with CanL presented a lower proportion of Anti-Leish-PC in their RP than in PALS. Likewise, dysproteinemia was related to RP and PALS plasmacytosis, and a more severe clinical profile.

## 1. Introduction

Zoonotic Visceral Leishmaniasis (ZVL) is a parasitic disease with a great impact on public health. It is present in several countries around the world, including Brazil [1]. Dogs can be affected by visceral leishmaniasis (CanL), and are an important source of infection for humans. In dogs, the disease can be severe, with progressive weight loss, onychogryphosis, alopecia, lymphadenopathy, hepatosplenomegaly, and death [2].

The spleen is an important secondary lymphoid organ which is involved in all cases of ZVL. Among the main histological changes observed in this organ during ZVL, the following stand out: hyperplasia followed by structural disorganization and the atrophy of lymphoid follicles, a decreased number of follicular dendritic cells, and red pulp (RP) plasmacytosis [3]. The alterations in splenic histology are usually associated with a high parasite load and more severe disease [4,5,6], whereas the preservation of spleen architecture was associated with less severe disease [7]. 

Plasma cells are final differential stages of activated B cells, and are primarily responsible for the production and secretion of antibodies [8]. Although many plasmablasts are produced in the spleen, under non-pathological conditions they do not remain in the organ. They emigrate to bone marrow, where a few long-lived plasma cells find their niche. In ZVL, an elevated amount plasma cells accumulates in the splenic RP [9]. At least two phenomena may contribute to spleen plasmacytosis in ZVL: (1) specific or polyclonal (not specific) B cell activation by *Leishmania* or coinfecting pathogens [10], and the homing and extended survival of otherwise short-lived plasma cell induced by BAFF, APRIL, and CXCL-12 [11,12]. Although the plasma cell density in the splenic RP correlates with the levels of hypergammaglobulinemia observed in ZVL, little is known about whether the fraction of the plasma cells present in spleen is committed with anti-*Leishmania* specific antibody production [13,14]. 

Thus, in this work, we used a modified immunohistochemistry (IHC) technique to identify *Leishmania*-specific antibody-producer plasma cells in different spleen compartments.

## 2. Results

### 2.1. General Characteristics of the Dogs Used in the Study

All of the dogs with CanL presented at least one clinical singn of disease classified into mild (2/8) or severe (6/8) intensity. The five most common signs of disease were conjunctivitis, pinna crust, emaciation, mucous hipocorated and onychogryphosis. Unfortunatelly, we obtained serum biochemistry from 5 out of the 8 dogs with CanL. Neverthenless, these animals presented an increased concentration of total proteins with low concentrations of serum albumin, resulting in dyproteinemia, with a median albumin/globulin ratio (0.4 [0.3–0.5]) substantially lower than that observed in the control animals (1.4 [1.1–1.4], Table 1).

### 2.2. Histological Changes of the Spleen

**Figure 1:**
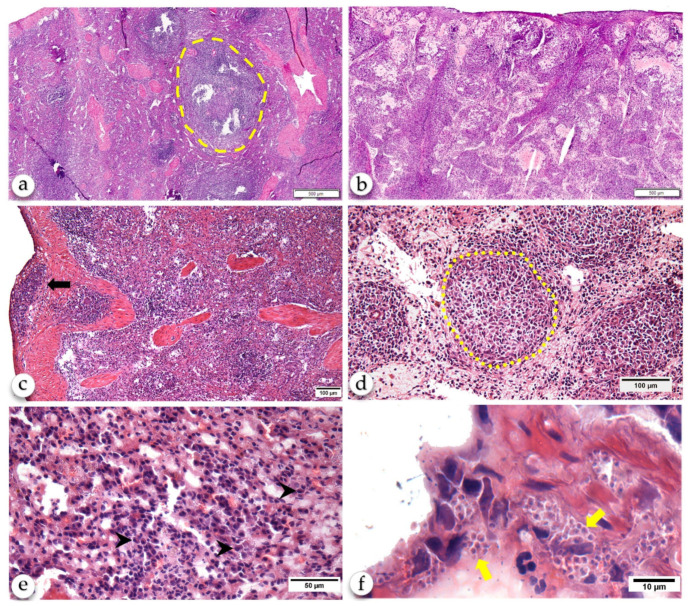
Spleen of dogs with CanL: (**a**) Spleen type 1. Note that the PALS, the lymphoid follicle (surrounded by a yellow dashed line), and the germinal center are well preserved. (**b**) Spleen type III. Note the disorganization of the WP compartments and lymphoid follicle atrophy. (**c**) Perisplenitis: chronic inflammatory infiltrate in the spleen capsule (black arrow). (**d**) RP granuloma surrounded by a yellow dotted line. (**e**) Numerous plasma cells in the splenic RP (black arrow heads). (**f**) Parasitized cells in RP; we can observe amastigotes (yellow arrows) of *L. infantum*. Hematoxylin and eosin staining.

The animals of control group presented a normal spleen classified as type 1 [3]. They presented well-defined compartments of RP and WP, including the PALS, MZ, and lymphoid primary and reactive lymphoid follicles presenting a germinal center and a well-delimited mantle zone. The RP presented a predominance of lymphocytes and macrophages with few plasma cells. The dogs with CanL presented perisplenitis (2/8), amastigote-containing macrophages (4/8) and granuloma (1/8) in the RP, type 3 (disorganized) WP (3/8) and lymphoid follicle atrophy (2/8). The dogs without CanL presented none of these changes. In comparison with the control healthy dogs, the animals with CanL had intense RP plasmacytosis (*p* = 0.0121, Mann-Whitney) (Table 2 and Figure 1). As expected, plasma cells were distributed in the PALS and RP. There was a trend towards a weak association between the density of plasma cells in the PALS and RP.

### 2.3. Anti-Leishmania-Specific Antibody-Producing Cells in the Spleen

**Figure 2:**
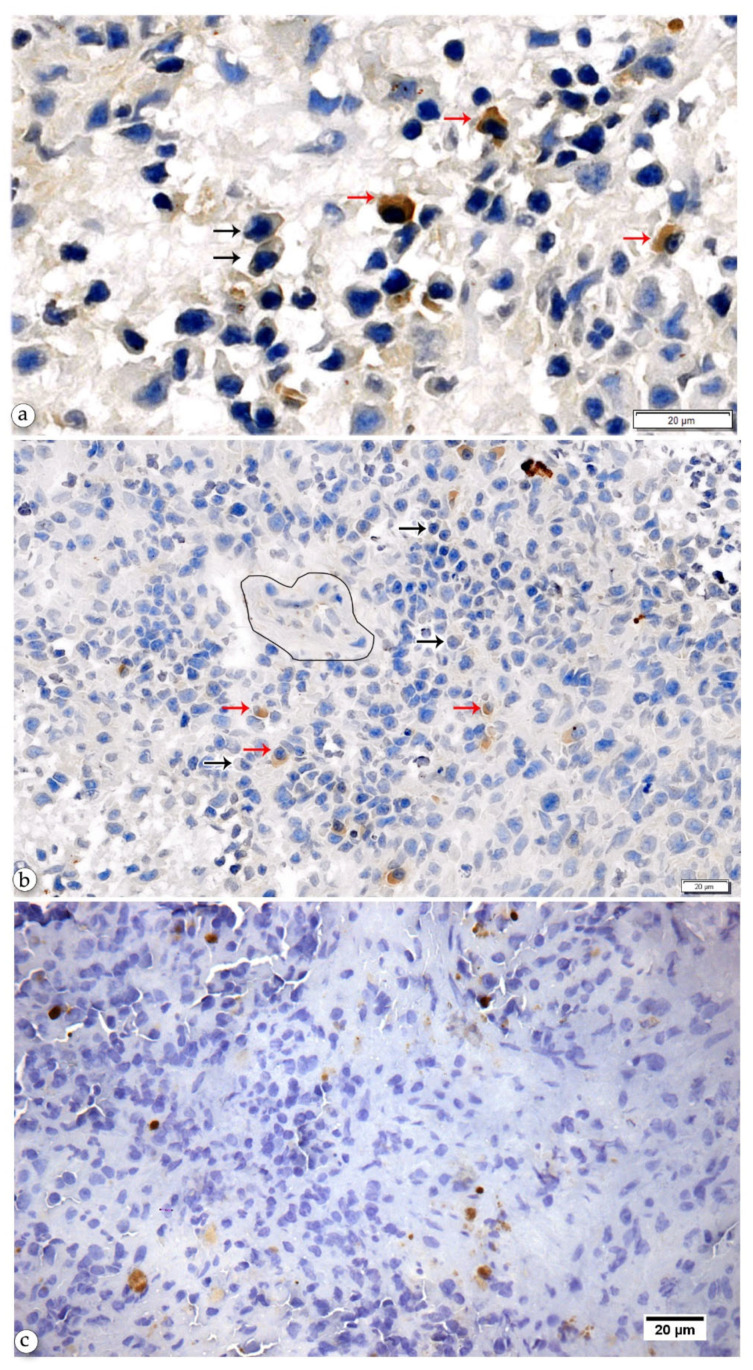
Modified IHC for the detection of anti-Leishmania specific antibody-producing plasma cells: (**a**) Leishmania antigen-positive (red arrow) and negative (black arrow) plasma cells in the splenic RP of dogs with CanL. (**b**) Leishmania antigen-positive (red arrow) and negative (black arrow) plasma cells in the PALS (around the central arteriole, surrounded by a black line). (**c**) No Leishmania antigen-positive plasma cells were in the RP of the control dogs; there was only iron pigment.

Anti-*Leishmania* antigen-specific antibody-producing plasma cells (Anti-Leish-PC) were present mostly in the spleens of animals with CanL. These cells were distributed in the RP and PALS (Figure 2). The mean of the percentage of Anti-Leish-PC tends to be higher in the PALS (59 ± 26) than in the RP (23 ± 13) (Table 3). We did not find plasma cells in other areas of the WP.

**Figure 3:**
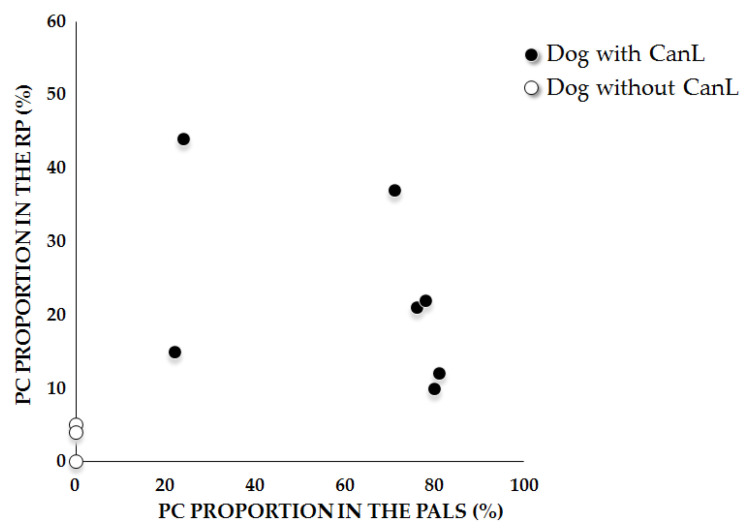
Distribution of the percentage of Anti-Leish-PC in the PALS and RP of dogs with or without CanL. Each point represents the median of the percentage for each animal (white dots = control animals, black dots = dogs with CanL).

As PC accumulated, the density of Anti-Leish-PC also increased in the RP and in PALS. However, there was a trend towards a negative association between the percentage of Anti-Leish-PC in the RP and PALS (Figure 3).

### 2.4. Non-Specific Plasma Cells may Contribute to the Severity of CanL

**Figure 4:**
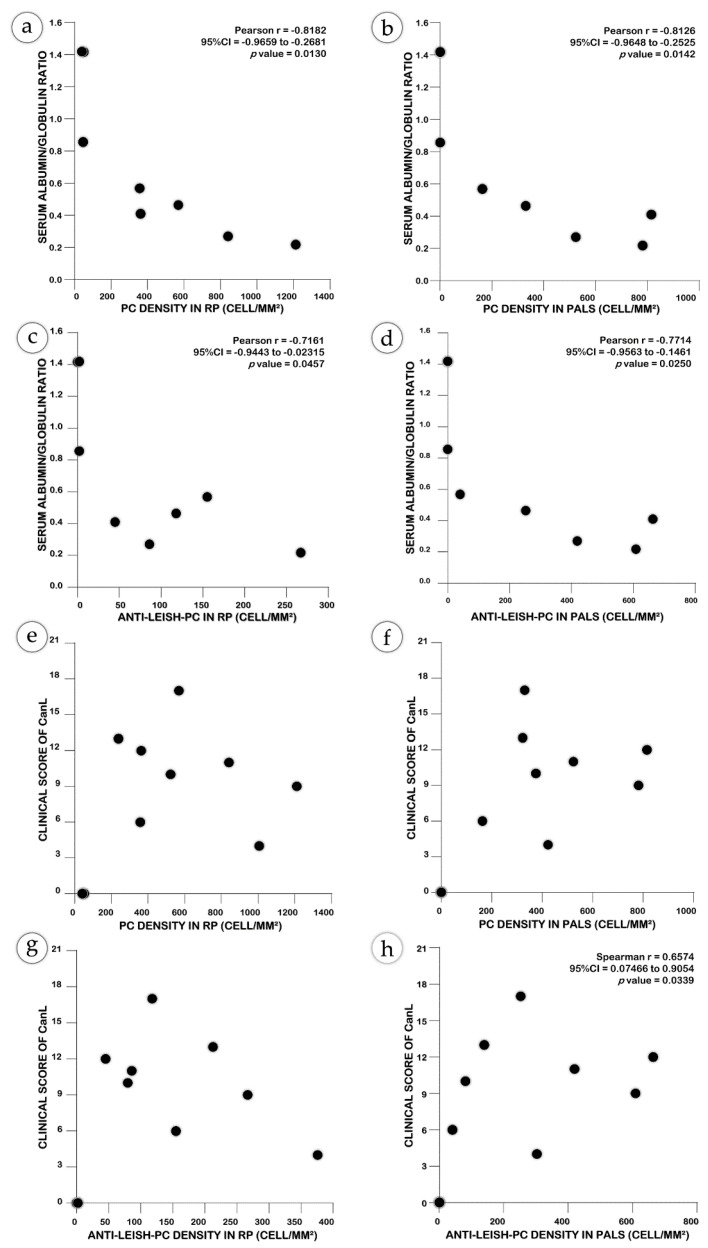
Clinical and laboratorial associations of splenic plasmacytosis: (**a**–**d**) with serum dysproteinemia; (**e**–**h**) with clinical scores of CanL. Strong associations can be observed between the density of PC and Anti-Leish-PC in the RP (**a**,**c**) and in the PALS (**b**,**d**). The Anti-Leish-PC density in the PALS becomes high as the clinical score increases (**h**). The clinical score tended to be high in dogs with spleen plasmacytosis; however, this association was not always statistically significant according to Pearson’s and Spearman’s correlation scores.

The density of the PC and of Anti-Leish-PC in the RP or PALS correlated with the dysproteinemia present in the serum of the dogs (Figure 4a–d). Furthermore, animals with high clinical scores also presented a high PC or Anti-Leish-PC density in the red or white pulp (Figure 4e–h).

## 3. Discussion

In a previous study by Silva-O’hare and colleagues (2016), it was shown that spleen plasmacytosis correlates with serum dysproteinemia in animals with CanL [9]. Herein, we extended this observation, showing that the plasma cell density in the RP or in PALS in isolation, correlates with serum dysproteinemia in dogs with CanL. We also revealed the distribution of PC-producing antibodies which are reactive with *Leishmania* antigens in the different spleen compartments in dogs with different stages of CanL, and showed that these Anti-Leish-PC predominate in PALS and RP, and were rare or absent in other spleen compartments. 

Although plasmacytosis is a remarkable finding in ZVL, in humans and in dogs we know little about the role played by these cells in the disease. In systemic lupus erythematous, these cells contribute to the production of autoantibodies which are responsible for the main manifestations of the disease [15]. It has also been shown that immunoglobulins produced in excess can accumulate in tissues, triggering a non-specific inflammatory response through the Fc portion of the molecules [16,17,18]. There are few studies that report the presence of autoantibodies in patients with ZVL; these antibodies may be produced by polyclonal activation, epitope spreading or mimicry between *Leishmania* and host molecules [19,20,21]. Furthermore, amyloidosis is reported in association with infection with some strains of *L. donovani* and *L. infantum* [22,23,24]. Herein, we showed that spleen plasmacytosis correlates with serum dysproteinemia, a marker of the severity of ZVL supporting a potential role of the antibodies produced by these cells in the pathogenesis of the disease. However, although ZVL can share some clinical characteristics with autoimmune diseases, studies are needed to better characterize the role of these antibodies in the disease, through specific or unspecific reactions.

Using a modified immunohistochemistry assay previously described by Mizutani and colleagues (2009), we were able to detect Anti-Leish-PC in the spleen of dogs [25]. As expected, the proportion of these cells was higher in the PALS than in the RP [26]. Because we used the surface antigens of promastigotes of *L. infantum*, plasma cells committed with other *Leishmania* antigens may not be represented in our survey. Nevertheless, the density of Anti-Leish-PC in the PALS or in the RP correlates with the serum dysproteinemia observed in dogs with CanL, supporting this hypothesis.

Additionally, we showed that animals with a high proportion (>70%) of Anti-Leish-PC in their PALS have more severe disease, and there is a trend toward a decrease in the proportion of Anti-Leish-PC in the RP. These animals also have a high density of PC in their RP. Some possible explanations can be given to explain this phenomenon: (1) restrictions may exists on the number of sites for PC transit in the outer PALS [27]; (2) it is possible that the inflammatory changes that promote the homing and extended survival of PC in RP [28,29] also take place in the PALS, impairing the transit of these cells to the RP; (3) polyclonal activation [12,20,30] and the antibody response to coinfections (which are frequent in severe cases of ZVL) [31,32] may contribute to the decrease in the proportion of Anti-Leish-PC in the RP. 

Among the limitations of this study, we highlight the small sample size and the use of promastigote surface membrane antigens. For this work, we were only able to obtain a small convenience sample kindly provided by another research group that worked in the field, before the COVID-19 pandemics. The fact that it deals only with promastigote surface membrane antigens did not allow us to explore a wider range of plasma cell reactivity with *Leishmania* (cytoplasmic or amastigote) antigens. Experiments aiming at the use of cytoplasmic and amastigote antigens, as well as methods of epitope recovery, are now in development in our laboratory. It is also our plan to expand the sample size for future studies.

Finally, to our knowledge, this is the first study to show the distribution of Anti-Leish-PC in the spleen compartments in ZVL. The modified IHC described herein can be used in further studies to characterize the role of plasma cells in the pathogenesis of severe forms of ZVL.

## 4. Materials and Methods

### 4.1. Ethical Statement

The project and procedures using animals were approved by the Ethical Committee for Animal Research of Instituto Gonçalo Moniz (IGM-FIOCRUZ, license nos. 021/2011 and 017/2015), and were conditioned according to Brazilian law 11.784/2008.

### 4.2. Animal and Specimens

Spleen samples from eight animals were obtained from the Zoonosis Control Center (ZCC) of Camaçari, state of Bahia, Brazil, an endemic area for ZVL. The dogs tested positive by ELISA for CanL, and were voluntarily delivered by their owners to the ZCC to be euthanized. As a control, specimens from three dogs without CanL were obtained from the Instituto Gonçalo Moniz kennel.

All of the animals were evaluated, before euthanasia, for the presence of any CanL clinical signs, scored from 0 to 21 as previously described [33]. Briefly, the nutritional status, mucosa color, dermatologic lesions, ocular dermatitis, conjunctivitis, spleen and lymph nodes size, were classified from 0 to 2, where 0 means normal or absent, and 2 means extensively present. Then, the values were summed up and the animals were classified as Subclinical (clinical score ≤ 3), Mild (4 ≤ clinical score < 7) and Severe (clinical score > 7). Furthermore, blood was collected for the albumin and globulin dosage. The dogs were sedated using an intravenous solution of cetamin + xylazine, and were euthanized by an intracardiac injection of supersaturated potassium chloride solution.

Three sections of spleen (1 cm × 1 cm × 1 cm) were collected and processed as described: the Section 1 was preserved in RNALater solution for *L. infantum* qPCR; the Section 2 was frozen, for histological analysis and modified IHC; the third was ground in complete Schneider medium (Schneider + 20% fetal bovine serum [FBS], Gibco, Waltham, MA, USA) and cultured in a B.O.D. incubator at 24 °C to evidence parasitism. 

### 4.3. Production of Biotinylated Soluble Leishmania Membrane Antigen (bSLMA)

*L. infantum* promastigotes (strain MHOM/BR2000/Merivaldo2) were cultured in vitro until the stationary phase of growth in complete Schneider medium (Schneider + 20% fetal bovine serum [FBS], Gibco, Waltham, MA, USA) in a B.O.D. incubator at 24 °C. The promastigotes were washed 3 times in phosphate buffered saline (PBS) at 1800× *g* at 4 °C for 10 min. They were resuspended at 6 × 10^7^ promastigotes/mL in a solution of 2 mg Biotin + 3% DMSO + PBS and incubated for 1 h at 37 °C, 5% CO_2_.

After incubation, the biotinylated promastigotes were washed 3 times with PBS + 5% FBS (1150× *g*, 4 °C, 10 min), resuspended in lysis buffer (NP40 + Tris HCl + EDTA, *v*/*v*) and shaken for 10 min, then centrifuged for 3 min (1150× *g*, 4 °C). All of the contents were transferred to a microcentrifuge tube and spun 15,700× *g*, for 5 min at 4 °C. The supernatant was collected and stored at −20 °C until it was used as a probe in modified IHC.

### 4.4. Modified Immunohistochemistry

In order to identify cells producing antibody anti-*L. infantum* in splenic tissue, we used a modified IHC technique, as previously described [34,35], with some adaptations. Briefly, frozen sections of 4–5 μm thickness were collected in charged slides, acclimated to room temperature for 5 min, and hydrated in distilled water. Endogenous peroxidase was inhibited by incubation with 0.3% H_2_O_2_ + methanol, for 30 min at room temperature. The antigen biding sites were retrieved using Proteinase K (5 μg/mL + 0.05 M Tris Buffed Salin, pH 7.6) for 15 min, and the sections were washed with PBS + 1% BSA. The sections were overlayed with 50μL bSLMA (1:100, in PBS + 1% BSA) and incubated for 1 h at room temperature. They were then incubated with Streptavidin-HRP 2μg/mL at room temperature for 1 h, washed 3 times, and the reaction was developed with 0.02% DAB + 0.006% H_2_O_2_ + 0.05 M Tris-HCl pH 7.6. The sections were counterstained with hematoxylin, dehydrated in gradient concentration ethanol, mounted, and examined under an optical microscope.

### 4.5. Histological Analysis and Cell Quantification 

Spleen sections stained with HE were analyzed under conventional microscopy by a pathologist, using the histological parameters previously defined [36]. Briefly, the spleen capsule was examined for the presence of inflammation or fibrosis. The cell populations present in the RP and WP were identified, and the main constituents were recorded. The presence of infected macrophages, granuloma or hyaline deposits in the germinal centers and fibrosis were recorded. WP was classified as proposed by Hermida et al (2018); briefly: Spleen type 1, with a well-organized WP, presenting a distinct lymphoid follicle (with well-defined germinal centers), mantle zone and PALS; Spleen type 2, with hyperplasic or hypoplastic lymphoid follicle, with faded boundaries of WP regions; Spleen type 3, moderately to extensively disorganized, with barely any or indistinguishable delimitation of the spleen compartments. In these, type 3 WP lymphoid follicles were frequently atrophic [3].

Hematoxylin and eosin-stained and modified IHC stained slides were scanned using a 40× objective of a Virtual Slide Microscopy VS120 (Olympus, Tokyo, Japan) and analyzed using Image-Pro Plus 7.0 software (Media Cybernetics, Rockville, MD, USA).

The total plasma cells counting was performed in five random fields of the RP of HE-stained slides. Only cells with a characteristic morphology of plasma cells (slightly acidophilic cytoplasm, a nucleus displaced to the periphery of the cell, and the clear area close to the nucleus corresponding to the Golgi complex) were counted [8]. The result was expressed as plasma cell/mm² for each animal.

A similar procedure was used for the estimation of the density of plasma cells positive for *Leishmania* antigens in IHC: Anti-Leish-PC were considered as cells with the morphological characteristics described above and stained in brown (DAB staining, Sigma-Aldrich, Saint Louis, MO, USA); plasma cells were considered as cells with a plasma cell morphology, but without brown staining. The results were expressed as the percentage of Anti-Leish-PC among all of the plasma cells. 

### 4.6. Statistical Analysis

The numerical data are presented in tables or graphs representing absolute numbers, means, medians or percentages, as indicated. The comparisons of the means or medians between the healthy and CanL dogs were performed using a *t*-test or a Mann-Whitney test. For comparisons between the percentages of positive cells in the RP and PALS, we used a Mann-Whitney test. Pearson’s and Spearman’s correlations were used to associate the serum albumin–globulin ratio or clinical score of CanL to other variants such as the PC density and Anti-Leish-PC density. The threshold for statistical significance was set at a *p* < 0.05. All of the graphics and calculi were created using software GraphPad Prism 8.0.

## Figures and Tables

**Table 1:** Clinical and laboratorial characteristics of the dogs with or without CanL used in the study of anti-*Leishmania* antibody-producing plasma cells in the spleen.

Parameters	With CanL N(%)	Without CanL N(%)
N (%)	8 (100%)	3 (100%)
Sex:		
Male	7 (88%)	0 (0%)
Female	1 (12%)	3 (100%)
Estimated age (year):		
<1	1 (12%)	0 (0%)
1 to 2	2 (25%)	0 (0%)
>3	5 (62%)	3 (100%)
Clinical score:		
Subclinic (≤3)	0 (0%)	3 (100%)
Mild (≥4 and <7)	2 (25%)	0 (0%)
Severe (≥7)	6 (75%)	0 (0%)
Clinical signs of disease:		
Conjunctivitis	7 (88%)	0 (0%)
Pinna crust	6 (75%)	0 (0%)
Emaciation	5 (63%)	0 (0%)
Mucous hipocorated	5 (63%)	0 (0%)
Onychogryphosis	5 (63%)	0 (0%)
Splenomegaly	4 (50%)	0 (0%)
Periocular dermatitis	4 (50%)	0 (0%)
Alopecia	4 (50%)	0 (0%)
Seborrheic dermatitis	4 (50%)	0 (0%)
Lymphadenomegaly	3 (38%)	0 (0%)
Muzzle depigmentation	3 (38%)	0 (0%)
Ear ulcer	2 (25%)	0 (0%)
Hyperkeratosis	2 (25%)	0 (0%)
Laboratory signs of infection/disease:		
Serum proteins ^1^:		
Total	7.6 [6.8–8.0]	5.8 [5.8–6.8]
Albumin	2.0 [1.7–2.5]	3.4 [3.4–3.5]
A/G ratio	0.4 [0.2–0.5]	1.4 [0.8–1.4]
Positive qPCR	6 (75%)	0 (0%)
Positive ELISA	8 (100%)	-
Positive Culture of spleen aspirate	8 (100%)	-

^1^ The values of the total protein, albumin, and albumin/globulin ratio (A/G ratio) are represented as medians, and the first and third quartile (inside square brackets). The other values are presented as absolute numbers, followed by a percentage.

**Table 2:** Histological characteristics of dogs with or without CanL used in the study of anti-*Leishmania* antibody-producing plasma cells in the spleen.

Histological Parameters	With CanL N (%)	Without CanL N (%)
Perisplenitis	2 (25%)	0 (0%)
Granuloma	1 (12.5%)	0 (0%)
Amastigotes	4 (50%)	0 (0%)
White pulp classification		
Type 1 (organized)	5 (62.5%)	3 (100%)
Type 3 (disorganized)	3 (37.5%)	-
Lymphoid follicle size		
Normal	5 (62.5%)	3 (100%)
Athrophied	2 (25%)	-
Hiperplasic	1 (12.5%)	-
Plasmacytosis (cell/mm^2^)		
Red pulp	639 ± 346 ^a^	44.7 ± 5.7 ^a^
PALS	467 ± 228	0

^a^ Different statistical means tested using a Mann-Whitney test, *p* value = 0.0121.

**Table 3:** Morphometric analysis of the plasma cells and Anti-Leish-PC in the spleen of dogs with and without CanL.

Spleen Type ^1^	Red Pulp			PALS		
	Total PC	Anti-Leish-PC	(%)	Total PC	Anti-Leish-PC	(%)
With CanL						
1	362	45	(12%)	815	663	(81%)
1	523	80	(15%)	375	81	(22%)
1	569	118	(21%)	330	252	(76%)
1	1212	267	(22%)	782	608	(78%)
1	357	155	(44%)	164	40	(24%)
3	1006	377	(37%)	424	302	(71%)
3	842	86	(10%)	524	419	(80%)
3	239	213	(89%)	323	139	(41%)
Mean ± sd	639 ± 346	168 ± 112	(23 ± 13%)	467 ± 228	313 ± 234	(59 ± 26%)
Without CanL						
1	41	2	(5%)	0	0	(0%)
1	52	0	(0%)	0	0	(0%)
1	47	2	(4.2%)	0	0	(0%)
Mean ± sd	47± 5.5	1.3 ± 1.1	(3 ± 2.6%)	0	0	(0%)

^1^ See Methods for definitions. The absolute number represents cell counts/mm².

## Data Availability

Not applicable.
